# Genotypic diversity of *Acanthamoeba* strains isolated from Chilean patients with *Acanthamoeba* keratitis

**DOI:** 10.1186/s13071-019-3302-5

**Published:** 2019-01-25

**Authors:** María Isabel Jercic, Carolina Aguayo, Mónica Saldarriaga-Córdoba, Laura Muiño, Stella Maris Chenet, Jaime Lagos, Antonio Osuna, Jorge Fernández

**Affiliations:** 1 Sección Parasitología, Instituto de Salud Pública de Chile, Av. Marathon 1000, Ñuñoa, CP 7780050 Santiago, Chile; 2 Departamento de Parasitología, Instituto de Biotecnología, Grupo de Bioquímica y Parasitología Molecular, Universidad de Granada, Campus Universitario Fuentenueva, 18071 Granada, Spain; 3 Subdepartamento de Genética Molecular, Instituto de Salud Pública de Chile, Av. Marathon 1000, Ñuñoa, CP 7780050 Santiago, Chile; 4 Centro de Investigación en Recursos Naturales y Sustentabilidad, Universidad Bernardo O’Higgins, Av. Viel 1497, CP 8370993 Santiago, Chile; 5 Facultad de Ciencias de la Salud, Instituto de Ciencias Biomédicas, Universidad Autónoma de Chile, El Llano Subercaseaux 2801, San Miguel, CP 8910060 Santiago, Chile; 6 Facultad de Medicina, Universidad de los Andes, Monseñor Álvaro del Portillo 12455, CP 7550000 Las Condes, Chile

**Keywords:** *Acanthamoeba* keratitis, T4 genotype, T11 genotype, T2 genotype, ASA.S1, DF3

## Abstract

**Background:**

*Acanthamoeba* spp. are the causative agents of a severe keratitis occurring mainly in contact lens wearers. The genus comprises more than 24 species that are currently divided into 20 different genotypes (T1-T20) according to sequence variations in the *18S* rRNA gene. The objective of this study was to identify the genotypes and sub-genotypes of *Acanthamoeba* isolates collected at the Parasitology Laboratory of the Public Health Institute of Chile, the only laboratory in the country where *Acanthamoeba* screening is performed. This is the first report of genotypic identification of clinical isolates of *Acanthamoeba* in Chile and one of the few in South America.

**Results:**

In this study, 114 *Acanthamoeba* isolates from 76 *Acanthamoeba* keratitis patients, obtained between 2005–2016, were genotyped. T4 was the predominant genotype; T2 and T11 genotypes, which are scarcely reported worldwide, were also identified in Chilean patients (one and two patients, respectively). This is the first report of T2 and T11 genotypes isolated from *Acanthamoeba* keratitis patients in South America. It is also the first report of the T2 genotype circulating in this continent. Analysis of the diagnostic fragment 3 region of the *18S* rRNA gene showed 24 T4 variants, with a predominance of the sub-genotype T4/A, followed by T4/B, T4/G, T4/C and T4/D. Bayesian analysis revealed three groups among the T4 variants: two well supported groups that included 12 and 7 sub-genotypes, respectively, and a weakly supported group that included 5 sub-genotypes. Most of the predominant T4 sub-genotypes belonged to the same group, which included 71.3% of the patients, while some minority variants lied mainly in the other two clusters.

**Conclusions:**

T2, T4 and T11 genotypes were predominantly isolated from the *Acanthamoeba* keratitis patients in Chile. Chilean predominant T4 sub-genotypes, which have also been reported worldwide, formed a separate cluster of the minority T4 variants. This study provides useful information about the predominant genotypes and subgenotypes that would be useful in selecting suitable strains to develop immunological and/or molecular diagnostic assays in Chile.

**Electronic supplementary material:**

The online version of this article (10.1186/s13071-019-3302-5) contains supplementary material, which is available to authorized users.

## Background

*Acanthamoeba* is a genus of ubiquitous free-living microorganisms that can cause *Acanthamoeba* keratitis (AK), a painful and severe sight threatening corneal disease. AK is especially prevalent among contact lens users, which corresponds to 85–88% of the AK cases [1]. Since the first report of AK in 1973, the number of AK cases has increased concomitantly with the growing number of contact lens users [2, 3]. In Chile, up to 18 patients are diagnosed per year at the Public Health Institute of Chile [4].

*Acanthamoeba* spp. are usually classified on the basis of the nuclear small subunit *18S* ribosomal RNA full gene sequence (*Rns*), which allows the differentiation of *Acanthamoeba* spp. into 20 genotypes (T1-T20) [5–7]. Since the complete *Rns* gene exceeds 2000 nucleotides, a 423 to 551 bp fragment named as “*Acanthamoeba* specific amplimer (ASA.S1)” within the *Rns* gene is used for genotyping *Acanthamoeba* spp. [8]. A small but highly variable region inside ASA.S1 designated as DF3 (Diagnostic Fragment 3, ≈ 240 nucleotides long) is also used to determine the genotype of an isolate [9]. Since the vast majority of AK cases worldwide are caused by *Acanthamoeba* genotype T4, information below the DF3 level is sufficient to establish a sub-genotype or variant [6, 10–12].

*Acanthamoeba* genotyping is not only necessary for taxonomic purposes but also for epidemiological and clinical studies. It also provides valuable information for the development of new diagnostic methods, helps in selecting appropriate strains for the obtention of antigen, protein profile characterization, etc. Furthermore, it is possible to identify correlations between the isolates and clinically relevant aspects such as virulence, drug susceptibility and/or clinical outcome [13].

All samples from patients with suspected AK in Chile are analyzed at the Public Health Institute of Chile (ISPCh). The samples are investigated by culture, and positive cultures are subjected to genotyping. The aim of the present study was to determine the prevailing genotypes of *Acanthamoeba* in AK infection in Chilean patients. To the best of our knowledge, this is the first study of this nature in Chile and one of the few performed in South America.

## Methods

### Collection of clinical specimens

The samples received for *Acanthamoeba* diagnosis at the ISPCh were processed as described previously [4]. Briefly, the samples were inoculated onto 2% non-nutrient agar plates overlaid with 100 μl of a liquid culture of *Escherichia coli* in Page’s solution (NNA-*E. coli*). The plates were incubated at 37 °C for seven days and examined daily under a conventional light microscope for the presence of trophozoites and/or cyst. All positive culture plates were routinely collected in Page’s solution, subcultured in 5 ml Petri dishes with NNA overlaid with live *E. coli*, and also diluted 1:2 with sterile glycerol for long term storage at -20 °C. Amoebas from subculture plates were harvested and rinsed three times in phosphate-buffered saline (pH 7.4) and then transferred to Eppendorf tubes for further molecular analysis. When the genotyping service was not readily accessible, the samples were stored frozen until the service became available. At that time, an aliquot of the frozen stock was inoculated onto NNA overlaid with live *E. coli* until growth was observed. Then, the samples of amoeba were collected and processed for molecular analysis as described above.

### DNA extraction and PCR amplification assay

*Acanthamoeba* trophozoites and cysts were lysed by thermal shock at 56 °C for 15 min with proteinase K; DNA was then extracted using a QIAamp DNA Mini Kit ® (Qiagen, Venlo, NLD) following the manufacturer’s protocol.

The ASA.S1 region in *Rns* was amplified using the *Acanthamoeba* genus-specific primers, forward JDP1 (5'-GGC CCA GAT CGT TTA CCG TGA A-3') and reverse JDP2 (5'-TCT CAC AAG CTG CTA GGG GAG TCA-3') as described by Booton et al. [10]. These primers amplified a fragment of approximately 500 bp. The PCR reactions were set up to a final volume of 50 μl, using 30 ng DNA, 1× amplification buffer [10 mM Tris-HCl (pH 9.0), 3.5 mM MgCl_2_], 2 mM of each dNTP, 20 μM of each primer, 1.25 U Taq DNA polymerase and distilled water to make the volume. Amplification cycles were performed in a GeneAmp System 2700 thermocycler (Applied Biosystems, Foster City, CA, USA). The PCR cycles were set up as follows: pre-denaturation step at 94 °C for 3 min and 35 cycles of denaturation at 94 °C for 30 s, 60 °C of annealing for 30 s and then 72 °C for 1 min, followed by a final elongation step of 72 °C for 5 min. T4 *Acanthamoeba* strains previously identified at our laboratory were used as a positive control and DNA-free water was used as a negative control. The amplicons were resolved by agarose-gel electrophoresis and visualized using ethidium-bromide.

### Sequencing and genotyping of strains

PCR products were purified using a DNA band purification kit (Omega Bio-Tek, Omega, Norcross, GA, USA), according to manufacturer’s instructions, and sequenced in both directions. The sequences of the PCR amplicons were obtained using the ABI PRISM BigDye Terminator Cycle Sequencing kit (Applied Biosystems, Foster City, CA, USA) with 5 pmol of JDP1 and JDP2 primers and a 310 ABI PRISM Genetic Analyzer.

### Alignment and data exploration

The sequences were assembled and edited with ALIGN, EditSeq and MegAlign (DNASTAR, Madison, WI, USA).

### Phylogenetic analysis

The Bayesian inference algorithms implemented in MrBayes v.3.0B4 were used to infer phylogenetic trees [14]. A total of 131 related nucleotide sequences of the *Rns* gene were selected considering an E-value close to zero and percentage identity > 30% [15].

The multiple alignment of the nucleotide sequences was performed in BioEdit and corrected in GeneDoc v.2.7.000 [16]. After including gaps to maximize alignments, the final number of nucleotide positions was 459 bp. For the phylogenetic analysis, we used *Acanthamoeba* T14 (*Acanthamoeba* sp. AF333609 and AF333607) genotypes as outgroups, following to phylogenetic tree obtained by Risler et al. [17]. In addition, we included genotypes T5 (*A. lenticulata* U94741), T2 (*Acanthamoeba* sp. AB425949; *Acanthamoeba polyphaga* ATCC30872, AY026244), T10 (*A. healyi* AF019070; *Acanthamoeba* sp. GU808320), T11 (*A. stevensoni* AF019069; *Acanthamoeba* sp. GU808311), T13 (*Acanthamoeba* sp. AY102615 and AF132136) and T16 (*Acanthamoeba* sp. AY026245 and GQ380408). The best-fitting model of nucleotide substitution was selected using the Bayesian information criterion implemented in the program MEGA v.7 [18, 19]. These results gave the best fit for the K2+Γ nucleotide substitution model [20].

We simultaneously executed three parallel MCMC runs, each for 30 × 10^6^ generations with four Markov chains (one cold and three hot chains). Each run was analyzed in Tracer to confirm that effective sample sizes (ESS) were sufficient for all parameters (posterior ESS values > 300) [21]. The nodes were considered supported if posterior probabilities (PP) > 0.95. The trees were visualized using FigTree v.1.1 available at http://tree.bio.ed.ac.uk/software/figtree/ [21].

## Results

### AK patients and samples

A total of 114 *Acanthamoeba* isolates, obtained from 76 patients, were genotyped between 2005–2016. The total number of samples analyzed by culture method during the period, the number of positive samples obtained, the number of samples genotyped, and the gender of the patients in each category, are shown in Table 1. The number of samples available for the analysis per patient ranged from one to four [4]. Forty-nine patients were positive for *Acanthamoeba* in more than one sample, while 27 patients showed a positive culture only from a single sample (Table 2). The *Acanthamoeba* isolates included in this study were obtained from both biological material (corneal scrape, biopsies and/or cotton swabs) and contact lens and its paraphernalia (contact lens boxes, lubricant and cleansing liquids).

**Table 1:** The number of samples and patients included in this study

	Tested^a^		Positive^b^		Genotyped^c^	
	No. of patients	No. of samples	No. of patients	No. of samples	No. of patients	No. of samples
Female	262	377	78	101	50	80
Male	156	247	51	64	26	34
Total	418	624	129	165	76	114

^a^The total number of samples and patients received at the ISP for *Acanthamoeba* screening between 2005–2016

^b^The number of samples and patients that were found positive by culture

^c^The number of positive samples and patients that were genotyped

**Table 2:** *Acanthamoeba* genotypes and accession numbers of the *Acanthamoeba* clinical strains isolated from Chilean patients with keratitis between 2005–2016

Patient ID	Sample code	Sex	Genotype	GenBank ID	Patient ID	Sample code	Sex	Genotype	GenBank ID
1	CHI1003	M	T4/E	JF702873	38	CHI6806	F	T4/G	JF702905
2	CHI1203	M	T4/A	JF702875	39	CHI7506	F	T4/N	JF702907
3	CHI1503	M	T4/M	JF702877	40	CHI9006	F	T4/C	JF702910
4	CHI1803	F	T4/B	JF702879		CHI9106		T4/C	MH100849
5	CHI3703	F	T4/A	JF702891	41	CHI9306	M	T4/O	JF702912
	CHI3803		T4/A	MH100821	42	CHI5007	F	T2	KX688012
	CHI4103		T4/A	JF702895	43	CHI6007	F	T4/A	KX688013
	CHI4203		T4/A	MH100825	44	CHI7007	F	T4/P	KX688014
6	CHI4003	F	T4/A	JF702894	45	CHI9007	F	T4/G	KX688015
7	CHI4503	M	T4/A	JF702897	46	CHI9507	M	T4/G	KX688016
8	CHI4703	M	T4/A	JF702898	47	CHI0608	F	T4/A	KX688017
9	CHI5303	F	T4/A	MH100830	48	CHI2808	F	T4/E	KX688018
	CHI5403		T4/A	JF702900	49	CHI3408	M	T4/A	KX688019
10	CHI56A03	F	T4/A	MH100834	50	CHI4708	M	T4/D	KX688020
	CHI56B03		T4/A	MH100835	51	CHI9608	M	T4/B	KX688021
	CHI5603		T4/A	JF702902	52	CHI1309	F	T4/R	KX688022
11	CHI2504	F	T4/Q	JF702882	53	CHI2809	M	T4/B	KX688023
12	CHI3804	F	T4/C	JF702893	54	CHI4309	F	T4/G	KX688024
	CHI4004		T4/C	MH100822		CHI4409		T4/B	KX688025
13	CHI5304	M	T4/C	MH100831	55	CHI7509	F	T4/A	KX688026
	CHI5404		T4/C	MH100832	56	CHI2810	M	T4/B	KX688027
	CHI5504		T4/C	JF702901	57	CHI5010	M	T4/F	KX688028
14	CHI5604	F	T4/A	JF702903		CHI5210		T4/A	KX688029
15	CHI5904	F	T4/D	JF702904	58	CHI5910	F	T4/A	KX688030
16	CHI7004	F	T4/D	MH100839		CHI6010		T4/G	KX688031
	CHI7204		T4/D	JF702906	59	CHI4011	M	T4/F	KX688035
17	CHI8204	F	T4/H	MH100844	60	CHI4411	F	T4/B	KX688036
	CHI8304		T4/H	MH100846	61	CHI7011	F	T4/V	KX688033
	CHI8404		T4/H	JF702908	62	CHI0112	F	T11	KX688037
18	CHI8804	F	T4/A	MH100847		CHI0312		T11	KX688039
	CHI8904		T4/A	MH100848		CHI0412		T11	KX688040
	CHI9004		T4/A	JF702909		CHI0212		T11	KX688038
19	CHI9304	F	T4/A	JF702911	63	CHI1812	M	T4/D	KX688034
20	CHI0405	M	T4/A	JF702872	64	CHI0615	F	T4/C	MH100807
21	CHI1805	M	T4/A	MH100814		CHI0715		T4/C	MH100808
	CHI1905		T4/A	JF702880	65	CHI1815	M	T4/F	MH100816
22	CHI2405	F	T4/A	JF702881	66	CHI2515	F	T4/C	MH100818
	CHI2505		T4/A	MH100817		CHI2615		T4/C	MH100820
	CHI2605		T4/A	MH100819	67	CHI5015	F	T4/B	MH100827
23	CHI2705	F	T4/A	JF702883		CHI5115		T4/B	MH100829
24	CHI3005	F	T4/A	JF702885	68	CHI6715	F	T4/B	MH100838
25	CHI3105	F	T4/A	JF702886	69	CHI0416	F	T4/E	MH100805
26	CHI3205	F	T4/G	JF702887		CHI0516		T4/E	MH100806
27	CHI3405	F	T11	JF702889	70	CHI0716	M	T4/A	MH100809
28	CHI3505	F	T4/M	JF702890	71	CHI0916	F	T4/K	MH100810
29	CHI3705	M	T4/A	JF702892		CHI1016		T4/K	MH100811
30	CHI4905	F	T4/A	JF702899	72	CHI4016	M	T4/X	MH100823
31	CHI1106	M	T4/A	JF702874		CHI4116		T4/S	MH100824
32	CHI1306	M	T4/D	JF702876		CHI4216		T4/W	MH100826
	CHI1506		T4/D	MH100812	73	CHI5016	F	T4/U	MH100828
33	CHI1606	F	T4/A	MH100813	74	CHI5416	M	T4/L	MH100833
	CHI1706		T4/A	JF702878		CHI6116		T4/L	MH100836
	CHI1806		T4/A	MH100815	75	CHI7016	F	T4/E	MH100841
34	CHI2806	F	T4/I	JF702884		CHI7116		T4/E	MH100842
35	CHI3206	M	T4/A	JF702888	76	CHI8116	F	T4/T	MH100843
36	CHI4706	F	T4/H	JF702896		CHI8216		T4/U	MH100845
37	CHI6406B	F	T4/J	JF702914					
	CHI6506		T4/J	MH100837					

*Abbreviations*: *M* male, *F* female

### Genotyping of the AK isolates and sequence analysis

The molecular identification of the *Acanthamoeba* isolates was confirmed by the detection of the PCR fragment of the expected size for all isolates (423–551 bp). The sequences of the ASA.S1 amplicons obtained in this study have been deposited in the GenBank database (accession numbers are shown in Table 2). By sequencing analysis of the DF3 region, 108 samples from the 73 patients were identified as genotype T4. Five strains from two patients were identified as *Acanthamoeba* sp. T11, while a single isolate obtained from a patient was identified as T2 genotype (Table 2).

The subgenus classification of the T4 isolates was done by the sequence analysis of a 54–69 bp long variable region of the DF3, which revealed the presence of 24 sequence types, herein referred to as T4/A to X. The alignment of a highly variable section of the DF3 fragments showed four new sequence types (T4/L, S, T and U) that have not been previously described (Additional file 1: Table S1).

T4/A was the most prevalent sequence type being identified in 41 (38%) samples isolated from 28 (38.4%) patients. Furthermore, this T4/A variant was most frequent among the corneal scrape isolates (19 out of 55 samples, 35.6%). The sequence type T4/B was identified in isolates from 8 patients; the sequence types C and D were obtained from five patients each; sequence types E, F and G were isolated from 4, 3 and 6 patients, respectively; sequence types H, M and U were found in samples from 2 patients each; while the remaining sequence types were identified in isolates from individual patients.

Most of the samples obtained from the same patient had the same type of T4 variant; however, in five cases (patient IDs: 54, 57, 58, 72 and 76) the samples presented different variants.

### Phylogenetic analysis

**Fig. 1:**
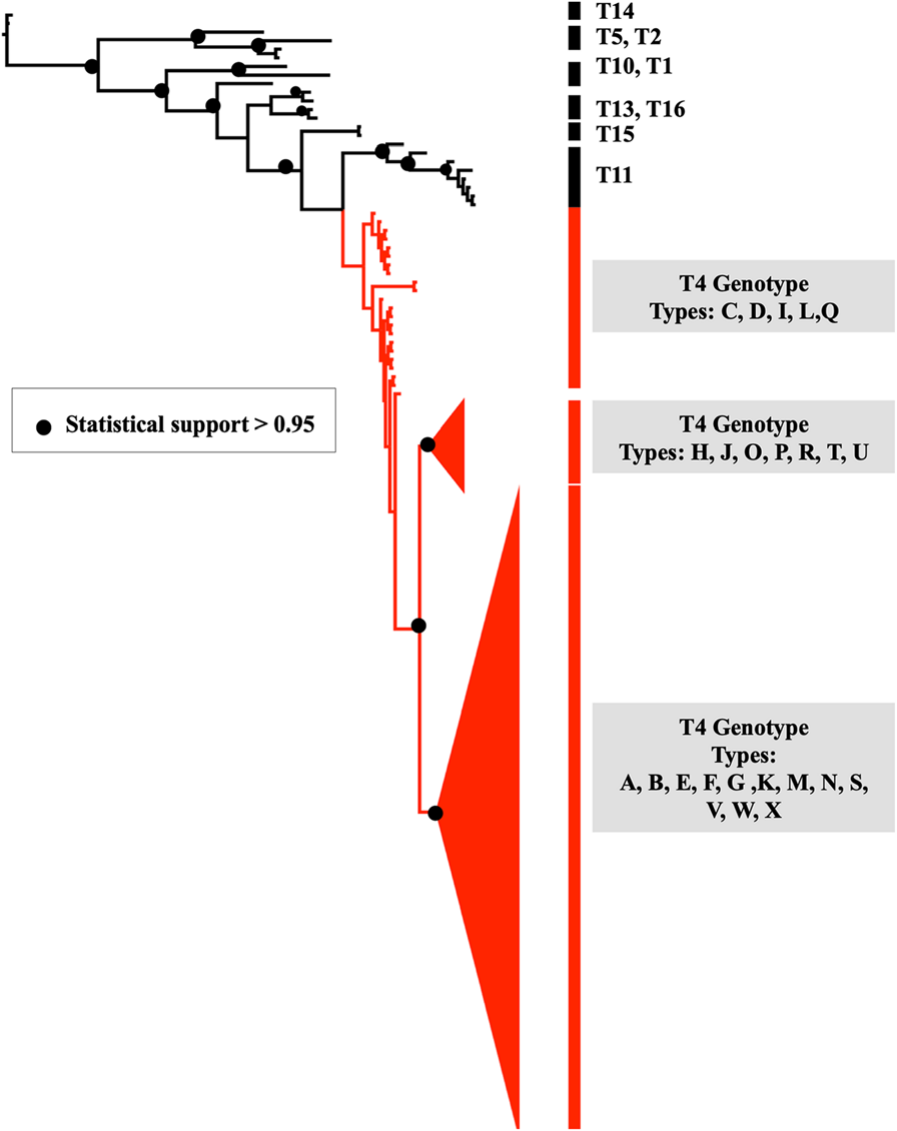
Phylogenetic tree based on the partial sequence of the *Rns* gene constructed using Bayesian inference analysis. The isolates from this study and reference *Acanthamoeba* strains are included

A total of 128 samples were included in this analysis (114 isolates obtained in this study and 14 reference strains; see methods). The phylogenetic tree constructed considering the partial sequence of the *Rns* gene (Fig. 1) was consistent with previous reports [17]. The tree showed well-supported clusters for each of the genotypes (PP ≥ 0.95). The sequences from patient samples used in this study were clustered according to genotypes T2 (*n* = 1), T11 (*n* = 5), and T4 (*n* = 108).

In relation to T4 genotype, the phylogenetic tree recovered three groups, two of them were well supported. The first group was weakly supported (PP = 0.52) and included the variants T4/C, D, I, L and Q. The second group (PP = 0.95) included variants T4H, J, O, P, R, T and U. Finally, the third group (PP = 0.95) included variants T4A, B, E, F, G, K, M, N, S, V, W and X.

## Discussion

We genotyped 69.1% of the *Acanthamoeba* strains isolated at the ISPCh between 2005 and 2016, while the rest failed to revive after freezing. T4 was the predominant genotype found in Chilean isolates (73 out of 76 patients), as reported worldwide [10, 12, 17, 22–24]. T4 is also the predominant genotype in environmental samples, followed by T5, both in Chile and other countries [9, 25, 26]. None of our clinical isolates corresponded to genotype T5, further corroborating the observation that this genotype is clearly underrepresented in AK cases [13]. In three other patients, T2 and T11 genotypes were found. Contrary to T4, these genotypes have rarely been found associated with AK [12, 27–30]. Furthermore, in South America, genotype T11 has previously been reported only from the environmental samples, so this is the first report of this genotype being isolated from AK patients; it is also the first report of genotype T2 circulating in this area [26, 31–33]. It is worth mentioning that the sequences of genotype T11 reported from the environmental strains in Chile are different from those isolated from AK patients [26].

Among T4 isolates, 24 different DF3 sequence types from 108 isolates were identified in this study, herein referred to as T4/A to X. When comparing our DF3 sequences with those isolated from the environmental sources in a sole study of this type performed in Chile, only 3 out of 13 environmental sequences were the same as of ours [26]. This finding suggests the existence of different degrees of virulence among genotype T4 strains, which warrants further investigation. Of our DF sequences, 38% belonged to the T4/A variant; the finding of this sequence variant as the most prevalent in Chile differs from that reported in other countries [10, 13, 22, 34]. Many of the remaining DF3 variants have previously been reported in more than 10 countries (T4E, F, G, I, N, O and V), while others have been reported in few countries, of even are described here for the first time. These data suggest the predominant worldwide distribution of some variants, along with some minor variants having a highly low distribution and, perhaps, weaker pathogenic properties. The Bayesian inference analysis further supports this classification, since most of the predominant and widely distributed variants were clustered together. Further studies should be conducted in order to elucidate what makes some variants more pathogenic than others. This information would also be valuable in order to select suitable strains for antigen production and development of diagnostic methods.

Finally, in most cases where more than one sample was available for culture, the same DF3 allele was isolated from all of them, following other authors [13]. In discordant cases (5 out of 27), it is possible to assume that the infecting variant is the one found in the corneal scrape or the biopsy. In that sense, patient no. 72 was a particular case, providing different DF3 sequences between two corneal scrapes taken from the same eye, suggesting a truly mixed infection. Unfortunately, the low number of patients showing different variants precludes the analysis of a possible correlation between source and variant, which could provide an approximation of the real pathogenic potential of the different variants isolated.

## Conclusions

We report for the first time the genotypes of AK causing strains circulating in Chile, obtained between 2005 and 2016. Chilean AK isolates were genotyped as T2, T4, and T11. 24 DF3 variants were identified within the predominant genotype T4. The Bayesian inference analysis showed that Chilean most prevalent T4 sub-genotypes, which have also been reported worldwide, formed a separate cluster of the minority or “local” T4 variants. This study provides useful information about the predominant genotypes and subgenotypes that would be useful in selecting suitable strains to develop immunological and/or molecular diagnostic assays in Chile.

## Additional file

Additional file 1: **Table S1.** Primary sequence alignment of a highly variable section of the DF3. (DOCX 26 kb)

## Abbreviations

AK: *Acanthamoeba* keratitis
ASA.S1: *Acanthamoeba* specific amplimer
DF3: Diagnostic fragment 3
dNTP: Deoxynucleoside triphosphate
ISPCh: Public Health Institute of Chile
NNA-*E. coli*: Non-nutrient agar plates overlaid with live *Escherichia coli*
PCR: Polymerase chain reaction
*Rns*: Nuclear small subunit 18S ribosomal RNA gene
