# Supplementary material for: Genotypic diversity of Acanthamoeba strains isolated from Chilean patients with Acanthamoeba keratitis
Source: Parasit Vectors. 2019 Jan 25;12:58. doi: 10.1186/s13071-019-3302-5 (PMC6346584; doi:10.1186/s13071-019-3302-5)
Supplement: Supplementary file 1 — Table S1. Primary sequence alignment of a highly variable section of the DF3. (DOCX 26 kb) [file 13071_2019_3302_MOESM1_ESM.docx]

Table S1. Primary sequence alignment of a highly variable section of the DF3

DF3 P/I^1^ Original Sequence alignment of the variable DF3 region Country in which the subgenotype

var denomination has been reported

....|....| ....|....| ....|....| ....|....| ....|....| ....|....| ....|....|

10 20 30 40 50 60 70

**T4/A** 28/41 T4/31[18] GGT-GCGGTC GTCCTTGGCG --TCGGTC-- TTTC---GGG GCCGG----C GCGGGGGCGG CTTAGCCCGG Chile, Iran, China, USA, France, Australia.

**T4/B** 8/9 T4/22[19] GGT-GCGGTC GTCCTTGGCG --TCGGTC-- TTTC---GGG GCCGG----C GCGGGGACGG CTTAGCCCGG Chile, Iran, Spain, China, USA, France, Australia.

**T4/C** 5/11 GGC-GCGGTC GTCCTTGGCG ---CGTTCGT GTTC---ACG CACGG-GCGC GCGAGGGCGG TTTAGCCCGG Chile, Iran, United Kingdom, Czech Republic, Austria.

**T4/D** 5/7 T4/33[18] GGC-GCGGTC GTCCTTGGCG -----TTCGT GTTC---ACG CACGA-GCGC G--AGGGCGG CTTAGCCCGA Chile, Iran, United Kingdom, Czech Republic, Austria.

**T4/E** 4/6 T4/6[14] GGTTGCGGTC GTCCTTGGCG --TCTC-GGT -TTC-----G GCCGGGG--C GCGGGGATGG CTTAGCCCGG Chile, Brazil, Iran, Poland, Malaysia, Taiwan, Germany, Italy,

Thailand, Philippines, China, Brazil, USA, Slovakia, Israel,

Korea, Argentina, Greece.

**T4/F** 3/3 GGC-GCGGTC GTCCTTGGCG --TCGGTC-- TTTC---GGG GCCGG----C GCGGGGGCGG CTTAGCCCGG Chile, Tanzania, Rwanda, Iran, Poland, Argentina, Germany, Italy,

Czech Republic, USA, Brazil, Slovakia, Malaysia, Austria,

China, Korea.

**T4/G** 6/6 T4/13[14] GGC-GCGGTC GTCCTTGGCG TCTCGGTC-- CTTC-ACGGG GCCGGGG--C GCGGGGGCGG CTTAGCCCGG Chile, Egypt, Iran, Brazil, Argentina, Venezuela, Turkey, Italy,

Poland, China, Philippines, USA, Spain, Taiwan, Switzerland,

France, Korea.

**T4/H** 2/4 GGC-GCGGTC GTCCTTGGCG T-TC-GTCAG CTTC---ACG GCCGGCGGGC GCGAGGGCGG TTTAGCCCGG Chile, Brazil, India.

**T4/I** 1/1 T4/24[20] GGC-GCGGTC GTCCTTGGCG ---CGTTCGT GTTC---ACG CACGG-GCGC GCGAGGGCGG CTTAGCCCGG Chile, Iran, United Kingdom, Spain, Taiwan, Brazil, Italy,

China, Peru, Austria, France.

**T4/J** 1/2 GGC-GCGGTC GTCCTTGGCG --T--GTCAG CTTC---ACG GCTGGCG--C GCGAGGGCGG TTTAGCCCGG Chile, Equatorial Guinea, France, Korea.

**T4/K** 1/2 GGC-GCGGTC GTCCTTGGCG TCTGTGTCCC TTTCAACGGG GGCATATGGC GCGAGGGCGG TTTAGCCCGG Chile, Spain.

**T4/L** 1/2 GGC-GCGGTC GTCCTTGGGG -----TTCGT GTTC---ACG CGCGA-GCGC --GAGGGGGG TTTAGCCCGG ---

**T4/M** 2/2 GGC-GCGGTC GTCCTTGGCG TC--GGTC-- TTTC---GGG GCCGG----C GCGGGGACGG CTTAGCCCGG Chile, Spain, Iran, France, USA.

**T4/N** 1/1 T4/32[18] GGC-GCGGTC GTCCTTGGCG TC--GGTC-- CTTC-ACGGG GCCGG----C GCGAGGGCGG CTTAGCCCGG Chile, Malaysia, United Kingdom, Japan, Germany, Iran, China, Turkey,

Poland, Brazil, USA, France, Switzerland, Italy, Sweden, Australia,

**T4/O** 1/1 GGC-GCGGTC GTCCTTGGCG ----TGTC-T CGGCTTCACG GCCGGGGCGC GCGAGGGCGG TTTAGCCCGG Chile, Malaysia, United Kingdom, Japan, Germany, Iran, China, Turkey,

Poland, Brazil, USA, France, Switzerland, Italy, Sweden, Australia.

**T4/P** 1/1 GGC-GCGGTC GTCCTTGGCG --T-TGTCGG CTTC---ACG GCTGGC-GGC GCGAGGGCGG TTTAGCCCGG Chile, Brazil, Poland, Iran, USA, Sweden.

**T4/Q** 1/1 T4/25[20] GGC-GCGGTC GTCCTTGGCG -----TTCGT GTTC---ACG CACGA-GCGC G--AGGGCGG TTTAGCCCGA Chile, Iran, United Kingdom, Turkey, China, USA, Korea, Greece.

**T4/R** 1/1 GGC-GCGGTC GTCCTTGGCG --T--GTCGG CTTC---ACG GCCGG--CGC GCGAGGGCGG TTTAGCCCGG Chile.

**T4/S** 1/1 GGT-GCGGTC GTCCTTGGCG --TCGGTT-- --TC-----C GCCGG----C GCGAGGGCGG CTTAGCCCGG ---

**T4/T** 1/1 GGC-GCGGTC GTCCTTGGCG --T--GTCGG TTTC---ACG GCCGGCG--C GAGAGGGCGG TTTAGCCCGG ---

**T4/U** 2/2 GGC-GCGGTC GTCCTTGGTG --T--GTCGG TTTC---ACG GCCGGCG--C GAGAGGGCGG TTTAGCCCGG ---

**T4/V** 1/1 T4/1[14] GGT-GCGGTC GTCCTTGGCG TCTCGGTC-- CTTC-ACGGG GCCGGGG--C GCGGGGGCGG CTTAGCCCGG Chile, Taiwan, India, Italy, China, USA, France, Thailand, Brazil,

Israel, USA.

**T4/W** 1/1 GGT-GCGGTC GTCCTTGGCG --TCGGTT-- --TC-----G GCCGG----G GCGAGGGCGG CTTAGCCCGG ---

**T4/X** 1/1 GGT-GCGGTC GTCCTTGGCG --TCGGTT-- --TC-----G GCCGG----C GCGAGGGCGG CTTAGCCCGG ---

^1^Number of patients vs. number of isolates for each DF3 variant. Sequences in grey have not been previously described.
